# Supplementary material for: A Histidine pH sensor regulates activation of the Ras-specific guanine nucleotide exchange factor RasGRP1
Source: eLife. 2017 Sep 27;6:e29002. doi: 10.7554/eLife.29002 (PMC5643099; doi:10.7554/eLife.29002)
Supplement: Figure 5—source data 1. [file elife-29002-fig5-data1.docx]

| **Protein** | RasGRP2cat and Rap1B |
| --- | --- |
| Space group | I 21 21 21 |
| **Cell Dimensions** |  |
| a, b, c (Å) | 139.1, 209.0, 335.9 |
| *α*, *β*, *γ* (˚) | 90, 90, 90 |
| **Data Collection** |  |
| Wavelength (Å) | 0.9795 |
| Resolution (Å) | 49.46 - 3.10 (3.15 - 3.10) |
| Rmerge | 0.148 (0.881) |
| I/*σ*(I) | 6.7 (1.5) |
| CC1/2 | 0.993 (0.699) |
| Completeness (%) | 100.0 (100.0) |
| Multiplicity | 4.5 (4.6) |
| **Refinement** |  |
| Number of unique reflections | 171499 |
| Rwork/Rfree (%) | 20.7/26.0 |
| Twinning operator and fraction | - |
| Average B value (Å) | 79.2 |
| Number of atoms | 34903 |
| **Stereochemistry** |  |
| Rmsd bond lengths (Å) | 0.003 |
| Rmsd bond angles (˚) | 0.56 |
| Ramachandran outliers (%) | 0.02 |
| Ramachandran favored (%) | 97.75 |
| PDB ID | 6AXF |

RasGRP4cat and HRas P 32 1 2

114.1, 114.1, 680.9

90, 90, 120

1.000

49.21 - 3.30 (3.37 - 3.30)

0.224 (0.612)

8.3 (1.9)

0.968 (0.521)

98.7 (94.0)

9.2 (4.8)

76133

22.8/26.0

(h, k, l) -> (k, h, -l) 0.40 90.5

23168

0.004

0.79

0.18

94.78

6AXG
